# Enhanced alveo pulmonary deposition of nebulized ciclesonide for attenuating airways inflammations: a strategy to overcome metered dose inhaler drawbacks

**DOI:** 10.1080/10717544.2021.1905747

**Published:** 2021-04-30

**Authors:** Hanan M. El-Laithy, Amal Youssef, Shereen S. El-Husseney, Nesrine S. El Sayed, Ahmed Maher

**Affiliations:** a Department of Pharmaceutics and Industrial Pharmacy, Faculty of Pharmacy, Cairo University, Cairo, Egypt; b Department of Pharmaceutics and Industrial Pharmacy, Faculty of Pharmacy, October University for Modern Sciences and Arts (MSA), Cairo, Egypt; c Department of Pharmaceutics, Egyptian Drug Authority, Cairo, Egypt; d Department of Pharmacology and Toxicology, Faculty of Pharmacy, Cairo University, Cairo, Egypt; e Department of Biochemistry, Faculty of Pharmacy, October University for Modern Sciences and Arts (MSA), Cairo, Egypt

**Keywords:** Aerosol, bronchial asthma, ciclesonide, nanolipid particles, nebulizer, pulmonary delivery

## Abstract

Ciclesonide (CIC), an inhaled corticosteroid for bronchial asthma is currently available as metered dose inhaler (CIC–MDI) which possesses a major challenge in the management of the elderly, critically ill patients and children. In this work, nebulized CIC nano-structure lipid particles (CIC-NLPs) were prepared and evaluated for their deep pulmonary delivery and cytotoxicity to provide additional clinical benefits to patients in controlled manner and lower dose. The bio-efficacy following nebulization in ovalbumin (OVA) induced asthma Balb/c mice compared to commercial (CIC–MDI) was also assessed. The developed NLPs of 222.6 nm successfully entrapped CIC (entrapment efficiency 93.3%) and exhibited favorable aerosolization efficiency (mass median aerodynamic diameter (MMAD) 2.03 μm and fine particle fraction (FPF) of 84.51%) at lower impactor stages indicating deep lung deposition without imparting any cytotoxic effect up to a concentration of 100 μg/ml. The nebulization of 40 µg dose of the developed CIC-NLPs revealed significant therapeutic impact in the mitigation of the allergic airways inflammations when compared to 80 µg dose of the commercial CIC–MDI inhaler (Alvesco^®^). Superior anti-inflammatory and antioxidative stress effects characterized by significant decrease (*p*< .0001) in inflammatory cytokines IL-4 and 13, serum IgE levels, malondialdehyde (MDA), nitric oxide (NO), TNF-α, and activated nuclear factor-κB (NF-κB) activity were obvious with concomitant increase in superoxide dismutase (SOD) activity. Histological examination with inhibition of inflammatory cell infiltration in the respiratory tract was correlated well with observed biochemical improvement.

## 1. Introduction

Bronchial asthma is a common chronic inflammatory disease of the respiratory tract and is one of the major health challenges in the twenty-first century that occurs in all countries regardless of the development level (Sherkawy et al., 2018). According to the World Health Organization (WHO), the number of patients suffering from asthma is 300 million people worldwide and is expected to increasingly rampant to 400 million, by 2025 (Hussain et al., 2018). It has serious medical, psychological and economic impact and accounts for ∼418,000 deaths a year globally. More seriously, childhood asthma is the leading chronic illness related to school absenteeism and disability (Hsu et al., 2016).

Asthma is characterized by shortness of breath, cough, chest tightness, and wheezing (Abdеlaziz et al., 2018). The common asthma triggers can be categorized as either allergic (pollens, dust, mold, and food allergens) or non-allergic including air pollution and irritants like cigarette smoke, respiratory infections, and strong odors (Ioachimescu & Desai, 2019). These triggers often aggravates and flare-up asthma leading to increased inflammatory cells infiltration especially eosinophil and interleukins causing muscle contraction and airway constriction (Qutubuddin et al., 2018).

Currently, inhaled corticosteroids (ICSs) are still the golden treatment that can effectively suppress the characteristic inflammation in asthmatic airways (Barnes, 2010). However, despite their beneficial clinical effects, their long-term treatment is associated with a well-known adverse events include possible growth retardation in children, osteoporosis, cataract formation, impaired immune response, and infections in the oropharynx. Such complications motivate developing a novel inhaled glucocorticoid with high local potency and minimal systemic absorption to improve safety profile (Leung et al., 2005). Therefore, ciclesonide (CIC), a new-generation non-halogenated gluco-corticosteroid with high local anti-inflammatory properties and no oral bioavailability was approved in the European Union in 2005–2006 for the treatment of asthma in adults and adolescent (Vogelmeier et al., 2011). CIC is an inactive prodrug and converted to its active metabolite, desisobutyryl-ciclesonide (des-CIC), in lung tissue by airway specific esterase (Boulet et al., 2007). Des-CIC has a 100-fold greater relative glucocorticoid receptor binding affinity than CIC which has almost no affinity for this receptor. In addition, des-CIC is lipophilic and readily conjugates to fatty acids at a hydroxyl group at C21 ensuring long-lasting anti-inflammatory effect on the lung and any absorbed portion is highly bound to plasma proteins (99%), leaving <1% of free drug in the systemic circulation, hence potentially minimizing systemic adverse effects (Leung et al., 2005). Although the commercial prevalence of CIC metered dose inhaler (MDI) yet, its clinical efficiency is limited by many errors such as poor coordination of aerosol generation with patient inhalation (Labiris & Dolovich, 2003). Other critical errors include quick breathing, stopping inhalation after firing the MDI and patient failing to hold the breath for further 6–10 s can all reduce lung deposition (Sanchis et al., 2013). The use of ethanol as co-solvent to improve the poor solubility of CIC in hydrofluroalkane (HFA) propellant used in the commercial product is another critical concern. Reduction in the formulation vapors pressure required for automation upon ethanol addition was reported with consequent increase in the mass median aerodynamic diameter (MMAD) and a decrease in the fine particle fraction (FPF) were inevitably impair the system performance and decrease delivered CIC to the lung (Myrdal et al., 2014; Zhu et al., 2015).

In addition to the above-mentioned obstacles, patients with severe asthma exacerbation pose special challenges to use MDIs as it requires deep, slow and steady inhalation which is very difficult with severe airway obstruction (Price et al., 2011). Therefore, the global initiative for chronic obstructive lung disease and for patients with low inspiratory force recommends nebulizers rather than DPIs or MDIs because the aerosol in nebulizers is continuously produced, and the aerosolized particles enter the airways in a repeated, slower, and humidified inspired air; therefore, the central airways are filled with a more uniformly distributed particles (Kamimura et al., 2012) while the patient is using tidal-volume breathing with minimal coordination and effort during inhalation (Dhand et al., 2012). Not only but also, larger doses of different compatible drug solutions can be mixed and nebulized together easily. Therefore, nebulizers are preferable for pediatric, elderly, ventilated, and unconscious patients (Ibrahim et al., 2015).

Clinically, asthma control still remains deficient owing to the inflammations located in the small airways, the major site of airflow limitation in asthma, and the relative inaccessibility of this lung region by conventional ICS which preferentially deposited in the larger airways even if high doses of ICS are prescribed (Berry et al., 2005). Therefore, the endeavors of targeting small peripheral airways could benefit to a great extent from the nanotechnology for designing CIC nanoparticle of suitable aerodynamic properties for small airways nebulization and better optimum asthma control (Usmani, 2015).

Nanostructured lipid particles (NLPs), a second generation of solid lipid nanoparticles, is a solid particulate carrier system made of solid outer layer entrapping oil core, therefore, allowing higher payload capacity of lipophilic drugs (Patlolla et al., 2010). Recently, aerosolizable NLPs have attracted much attention for the treatment of respiratory tract diseases owing to their vital potential to overcome intrinsic challenges encountered by the macrophagic lung clearance (Pardeike et al., 2011).

Based on literature review, only one report (Fu et al., 2018) has been attempted to provide pulmonary delivery of CIC till current date, but with limited success due to the obtained large MMAD∼6 µm of the prepared CIC suspension which promoted the majority of drug deposition in large conducting airways and oropharyngeal region. The higher amount of drug recovered in the gastrointestinal tract due to mucociliary clearance and complete removal of CIC suspension from the airways within 2 h is another miscarriage.

Thus, the objective of the current study was to explore the feasibility of encapsulating CIC into safe, biodegradable, biocompatible, and bioadhesive NLPs to the airways mucosal surface using more uniform nebulizable mode to elude mucociliary lung transit and provide deep local prolonged CIC therapeutic effect with less dosing frequency and better patient compliance (Pardeike et al., 2011; Weber et al., 2014). To confirm the efficacy of the developed CIC nano-structure lipid particles (CIC-NLPs), it was tested on ovalbumin (OVA)-induced asthma model in Balb/c mice. The anti-inflammatory and anti-oxidative stress effects as well as the histopathological changes were assessed and compared to Alvesco^®^, a commercially available MDI product.

## 2. Materials and methods

### 2.1. Materials

Compritol^®^ 888 ATO, gelucire 44/14, precirol ATO 5, capryol 90, labrafac lipophile WL1349, and lauroglycol FCC were kindly donated by Gattefossé (Lyon, France). Glyceryl monostearate was obtained from Daejung Chemicals (Seoul, Korea). Miglyol 812 was obtained from Sasol GmbH (Hamburg, Germany). Lecithin (70% phosphatidyl-choline) was purchased from Lipoid (Ludwigshafen, Germany). Tween 80, ciclesonide CRS, ovalbumin (OVA grade V), aluminum hydroxide, pyrogallol were purchased from Sigma-Aldrich (St. Louis, MO). Dimethyl sulfoxide and methanol of HPLC grade were provided by Merck (Darmstadt, Germany). All other chemicals and reagents were of analytical grade and used without further purification.

### 2.2. Methods

#### 2.2.1. Lipid screening

Four oils (capryol 90, labrafac, lauroglycol, and Miglyol812) and four solid lipids (precirol, compritol, glyceryl monostearate, and gelucire 44/14) were screened for maximum CIC solubility in each lipid. The solubility of CIC was estimated by dissolving excess amount of CIC in 2 ml liquid lipid/or adding 2 mg drug increments to the melted solid lipid (5–10 °C above melting temperature of each solid lipid) on a magnetic stirrer (MSH 420, BOECO, Hamburg, Germany) at 120 rpm for 24 h to yield a clear solution. The solubility of CIC in solid lipids was examined visually for the presence or absence of drug crystals. The maximum amount of the drug that remained soluble and beyond which precipitation took place was regarded as the solubility of the drug (Shevalkar & Vavia, 2019). Solubility in liquid lipid was estimated after separating the undissolved CIC by centrifugation at 15,000 rpm, at 25 °C for 20 min and the supernatant was separated and diluted with methanol. The concentration of the dissolved CIC was quantified using 1100 Agilent HPLC system with a UV detector (Agilent VWD G1314A, Santa Clara, CA). Chromatographic separation was made on Phenomenex^®^ C18column (250 mm × 4.6 mm, 10 µm) maintained at ambient temperature. The mobile phase composed of a binary mixture of HPLC grade absolute ethanol and deionized water at a ratio of 70:30 (v/v) filtered through 0.2 μm membrane filter, degassed before use and delivered at flow rate of 1 ml/min. Samples of 20 μl were injected and effluents were monitored at 242 nm (Elkady & Fouad, 2011). Linear correlation between peak area and CIC concentration was obtained within the concentration range of 10–1000 µg/ml (*r*^2^=0.998). All measurements were performed in triplicate and the results were expressed as mean values (µg/ml)±SD.

#### 2.2.2. Selection of solid and liquid lipid ratio

To test the miscibility between the best CIC solubilizing solid and liquid lipids, different ratios (9:1, 8:2, 7:3, 6:4, and 5:5) of lipid mixtures were stirred at 120 rpm for 1 h at 65, 80 °C for precirol and compritol, respectively. The lipid mixture was then cooled to congeal at room temperature (25 ± 1 °C). 24 h after solidification, a sample of the lipid mixture was smeared onto filter paper and observed visually for the existence of any oil droplets, which would indicate immiscibility between the solid and the liquid lipids (Mendes et al., 2013). Moreover, in order to obtain information on recrystallization of CIC from the lipid mixture, a drop of the melted drug–lipid mixture was inspected for the presence of drug crystals by light microscopy (Olympus CX31, Tokyo, Japan) (Kovačević et al., 2020).

#### 2.2.3. Experimental design

A 2^4^ factorial design was applied to study the effect of four critical independent formulation variables on the characteristics of the developed CIC-NLPs. (A) The solid:liquid lipid ratio (7:3, 9:1) and (B) the homogenization speed (10,000 and 20,000 rpm) at two levels (low and high) were screened as numerical variables whereas (C) solid lipid type (compritol and precirol), and (D) liquid lipid type (labrafac and miglyol) were evaluated as categorical factors. Other parameters, such as homogenization time, drug and surfactant concentrations were set at fixed values. A total of 16 runs were attempted using Design-Expert^®^ software (version 9, Stat-Ease Inc., Minneapolis, MN) and particle size (PS) (Y1), polydispersity index (PDI) (Y2), and entrapment efficiency (CIC-EE%) (Y3) as dependent responses were considered the critical attributes of the final target NLPs profile (Table 1). The polynomial equation generated from the experimental design is given below:

(1) $$Y=\;X_{0}+X_{1}A+X_{2}B+X_{3}C+X_{4}D+X_{12}AB+X_{13}AC+X_{14}AD+X_{23}BC+X_{24}BD+X_{34}CD\;$$

where *Y* is the dependent variable, *X*_0_ is the intercept, *X*_1_, *X*_2_ are the regression coefficients for the polynomial equations, *A*, *B*, *C*, *D* are the main effects, and the terms *AB*, *AC*, indicate the interactions between two factors. Statistical analysis was performed using ANOVA at 95% confidence level (*p*< .05). Additionally, the optimum formula was selected on the basis of the desirability approach that combines all the responses into one variable to predict the optimum levels of the studied factors (least PS and PDI associated with both the highest EE% and % cumulative drug release as well). Desirability index (*D*) has a range between 0 and 1, where *D* of 0 indicates an unacceptable formulation, and *D* of 1 denotes the most desired one (El-Laithy et al., 2018).

**Table 1.** Effect of dependent factors on CIC-NLPs critical attributes (Y1–Y3) expressed as mean values ± SD, *n* = 3.

Sample name	Solid lipid conc. (%w/w)	Liquid lipid conc. (%w/w)	Speed (rpm)	PS (nm)	PDI	EE (%)	DL^a^ (%)
NLP1	Compritol (7)	Miglyol (3)	10,000	702.1 ± 12.39	0.54 ± 0.021	73.968 ± 0.048	6.907 ± 0.045
NLP2	Compritol (9)	Miglyol (1)	10,000	346 ± 7.92	0.267 ± 0.009	90.904 ± 1.27	8.502 ± 0.106
NLP3	Precirol (7)	Miglyol (3)	10,000	365 ± 12.23	0.083 ± 0.034	89.696 ± 1.92	7.915 ± 0.179
NLP4	Precirol (9)	Miglyol (1)	10,000	222.6 ± 4.52	0.232 ± 0.019	93.316 ± 0.24	9.449 ± 0.020
NLP5	Compritol (7)	Labrafac (3)	10,000	626.65 ± 13.98	0.573 ± 0.027	85.511 ± 0.29	7.69 ± 0.092
NLP6	Compritol (9)	Labrafac (1)	10,000	231 ± 13.81	0.249 ± 0.017	90.838 ± 0.778	8.49 ± 0.049
NLP7	Precirol (7)	Labrafac (3)	10,000	670 ± 9.79	0.146 ± 0.031	86.683 ± 1.916	8.124 ± 0.027
NLP8	Precirol (9)	Labrafac (1)	10,000	380 ± 12.56	0.053 ± 0.018	91.092 ± 0.033	8.923 ± 0.171
NLP9	Compritol (7)	Miglyol (3)	20,000	1694 ± 7.07	0.806 ± 0.047	78.695 ± 1.601	7.235 ± 0.185
NLP10	Compritol (9)	Miglyol (1)	20,000	846.65 ± 13.37	0.831 ± 0.031	84.546 ± 0.035	7.514 ± 0.138
NLP11	Precirol (7)	Miglyol (3)	20,000	1003.7 ± 17.17	0.739 ± 0.036	87.365 ± 0.078	8.255 ± 0.02
NLP12	Precirol (9)	Miglyol (1)	20,000	742.8 ± 19.79	0.721 ± 0.017	90.546 ± 0.094	8.4010.21
NLP13	Compritol (7)	Labrafac (3)	20,000	1214.1 ± 14.54	0.774 ± 0.024	88.444 ± 0.145	8.351 ± 0.02
NLP14	Compritol (9)	Labrafac (1)	20,000	602 ± 13.81	0.946 ± 0.033	91.97 ± 0.102	9.016 ± 0.145
NLP15	Precirol (7)	Labrafac (3)	20,000	665 ± 4.01	0.674 ± 0.028	88.342 ± 0.053	8.301 ± 0.087
NLP16	Precirol (9)	Labrafac (1)	20,000	581 ± 12	0.631 ± 0.030	92.164 ± 0.778	9.171 ± 0.175

^a^ DL: drug load (not included in experimental design).

#### 2.2.4. Preparation of CIC-loaded NLPs

CIC-NLPs were prepared utilizing hot microemulsion technique (Mendes et al., 2019). Briefly, in a clear glass vial, the solid lipid (compritol or precirol at 7 or 9%) was melted 5–10 °C above the melting point of the respective lipid to produce internal imperfection for better accommodation of the liquid lipid (Elmowafy et al., 2018). CIC (0.01%) and lecithin as lipidic surfactant (0.5%w/w) were completely dissolved in liquid lipid (miglyol or labrafac at 1 or 3%) then added to molten solid lipid to form the lipid phase. At the same time, the aqueous surfactant phase of Tween 80 (2%w/w) in deionized water was preheated to the lipid temperature to avoid its recrystallization (Singh et al., 2019) and added drop wise to the lipid phase under thermostatic continuous stirring at 600 rpm (Banerjee et al., 2019) for 30 min. The resultant primary emulsion was further homogenized using an Ultra-Turrax T25 (IKA Labortechnik, Staufen im Breisgau, Germany) applying increasing intensities of 10,000 and 20,000 rpm for 5 min (Severino et al., 2012). Finally, the produced dispersions were cooled down to room temperature resulting in the formation of NLPs. Each formulation was prepared and characterized in triplicate.

#### 2.2.5. Particle size, polydispersity index, and zeta potential (ZP)

The mean PS, PDI, and ZP of CIC-NLPs were determined by photon correlation spectroscopy (PCS) technique using Malvern Zetasizer (Nano-ZS, Malvern Instruments, Malvern, UK). Prior to the measurements, each sample was diluted with de-ionized water (1:100) to avoid multiple scattering of the light caused by a high concentration of particle (Yousry et al., 2017). All measurements were performed at 25 ± 0.5 °C in triplicate.

#### 2.2.6. Encapsulation efficiency and drug loading (DL) capacity

Both EE and DL are determined indirectly by the separation of un-encapsulated CIC from the NLPs by centrifugation at 12,000 rpm at 4 °C for 30 min. The supernatant containing the un-encapsulated (free) drug was diluted with the mobile phase and quantified by the aforementioned HPLC method described in Section 2.2.1. The % of CIC-EE and DL were calculated by the following equations.

(2) $$\mathrm{EE}\;(\%)=\frac{\text{Total amount of CIC added in NLP}-\text{free amount of CIC}}{\text{Total amount of CIC added in NLP}}\times 100\;$$

(3) $$\mathrm{DL}\;(\%)=\frac{\text{Total amount of CIC added in NLP}-\text{free amount of CIC}}{\text{Total amount of lipid}}\times 100\;$$

#### 2.2.7. *In vitro* drug release

The *in vitro* release of CIC from NLPs was determined using the method reported by Elkady et al. (2020) to test release profile of drug particulate intended for pulmonary delivery in limited volume. The Franz diffusion cell with a diffusion area of 1.77 cm^2^ was used. The receptor compartment contained 7.5 ml of Gamble’s simulated lung fluid of pH 7.4 to mimic the interstitial fluid deep within the lung (Marques et al., 2011). The electrolytes composition and their order of mixing to avoid salt precipitation are presented in Supplementary table 1. Tween 80 (0.5% v/v) was added to maintain sink condition and the system was equilibrated at 37 ± 0.5 °C by a circulating water jacket. NLPs dispersion containing the equivalent of 40 µg CIC was dispersed in 200 µl of release media and placed in a dialysis membrane previously soaked overnight in the release medium before use, tightly tied from both ends and then loaded into the receptor compartment which was constantly stirred at 150 rpm with a small magnetic bar. At certain time intervals (0, 0.25, 0.5, 0.75, 1, 2, 4, 8, 12, 24, 36, and 48 h), 0.5 ml samples were aliquoted from the receptor compartment and replaced with equal volumes of fresh medium to maintain a constant volume. Samples were filtered using 0.22 μm filter and analyzed by HPLC method previously described. All release experiments were done in triplicates and the release data were fitted to various release kinetic models; zero, first, Higuchi diffusion as well as Hixson–Crowell and Korsmeyer–Peppas models (Ritger & Peppas, 1987).

#### 2.2.8. Thermal analysis

Differential scanning calorimetry (DSC) was applied for pure CIC, physical mixtures of NLP4 (precirol, miglyol, CIC) in addition to NLP4 formulation at a constant rate of 10 °C/min under atmospheric nitrogen using DSC analyzer (Shimadzu-DSC 50, Tokyo, Japan). The change in enthalpy was recorded over a temperature range from 30 to 400 °C (El-Laithy et al., 2019).

#### 2.2.9. Transmission electron microscopy (TEM)

The surface morphology of optimized CIC-NLP was examined using TEM microscope (JEM-1230, Jeol, Tokyo, Japan). One drop of diluted nanoparticle dispersion was deposited on carbon-coated copper grid (200 mesh) and negatively stained using 1% phosphotungstic acid. The grid was completely dried at ambient temperature and representative images of the sample were reported (Alam et al., 2018).

#### 2.2.10. *In vitro* aerodynamic properties

Aerodynamic properties of selected CIC-NLPs formulation were evaluated using eight-stage non-viable Andersen cascade impactor (ACI) (Copley Scientific Ltd., Nottingham, UK) equipped with filters on each stage (Hassanzadeh et al., 2017). CIC PS distribution was determined by connecting the jet nebulizer (3a Health Care S.r.l., Lonato, Italy) through its mouth piece directly to the induction port of cooled ACI, which placed with its plates *in situ* in a refrigerator at 5 °C for 60 min before use (Osama et al., 2019). The inhalation flow was adjusted at 28.3 l/min immediately after removing the ACI from the refrigerator according to the instrument specification for a nebulizer testing corresponding to human breathing rate (Smithmaitrie & Tangudomkit, 2018) by a vacuum pump (high-capacity pump mode, HCP) connected to critical flow controller model TPK (Copley Scientific Ltd., Nottingham, UK). CIC–NLP4 (3 ml) were placed in the nebulizer and aerosolized to dryness directly into the throat of the impactor. At the end of the experiment, the sample deposited into induction port, mouth piece, impactor stages (0–7) and filters were recovered with methanol, centrifuged at 12,000 rpm for 20 min and quantified by HPLC (Manconi et al., 2017). Aerodynamic parameters as MMAD, FPF, and geometric standard deviation (GSD) were automatically calculated from the cumulative mass distribution in the ACI using Copley Inhaler Testing Data Analysis Software (CITDAS; Copley Scientific, Nottingham, UK). The values obtained from three experiments were averaged as the results.

#### 2.2.11. Cell experiment

##### 2.2.11.1. Cell culture

Human alveolar type II epithelial cells (A549) was obtained from Nawah Scientific Inc. (Cairo, Egypt). Cells were grown in Dulbecco’s modified Eagle medium (DMEM; Thermo Fisher Scientific, Waltham, MA) pH 7.4, supplemented with 10% (v/v) of inactivated fetal bovine serum (FBS), 1% (v/v) penicillin/streptomycin and incubated at 90% humidity, 5% (v/v) CO_2_ atmosphere at 37 °C. The cells were allowed to grow until confluence using 0.1% trypsin solution in EDTA and seeded in plates for each experiments (Ungaro et al., 2012; Moreno-Sastre et al., 2016).

##### 2.2.11.2. *In vitro* cytotoxicity assay

The cytotoxicity and cell viability of CIC-NLPs were evaluated using sulfo-rhodamine B dye based assay (SRB; Sigma-Aldrich, St. Louis, MO). Exponentially growing cells were plated in a 96-well microtiter plates at a uniform cell density of 10,000 cells/well for 24 h before NLPs treatment. Cells were then treated with CIC-NLPs at various concentrations (0.01, 0.1, 1, 10, and 100 μg/ml) and incubated at 37 °C for 72 h. After drug exposure, negative control and treated cells were incubated and fixed at 4 °C with 10% trichloroacetic acid (TCA; Sigma-Aldrich, St. Louis, MO) for 1 h. The fixed cells were then washed with distilled water and kept at room temperature for air drying the wells overnight. Then, the cells were stained with 0.057% SRB solution for 1 h. The cells were washed with 1% acetic acid solution, dried for 10 minutes at 37 °C, and finally incubated in 10 mM Tris buffer (pH 10.5) and the absorbance was measured at 570 nm using a Fluostar Omega microplate reader (BMG Labtech, Ortenberg, Germany). Amount of protein synthesized was expressed as percentage of viability compared to the negative control (Kumar et al., 2016; Rashid et al., 2017).

#### 2.2.12. *In vivo* study

##### 2.2.12.1. Animals

Thirty-two female BALB/c mice weighing from 20 to 25 g (5–6 weeks old) were used. The animals were quarantined and acclimatized for one week prior to the initiation of experiments. During the quarantine period, animals were housed under environmentally controlled conditions (22 ± 2 °C; relative humidity, 50%±5%; 12 h light/dark cycle). Mice were fed a standard pellet chow and were allowed free access to food and water. This study was approved by Institutional Animal Care and Use Committee (CU-IACUC), Cairo University (CU III/F/59/19).

##### 2.2.12.2. Induction of asthma and treatment

Asthma was induced in mice through the intraperitoneal (i.p.) injection of 200 μl OVA-Al(OH)_3_ emulsion (10 μg OVA was emulsified with 100 μl of 1% Al(OH)_3_ in phosphate buffer (PBS) solution pH 7.4) on day 0 and day 7 of the experiment. The animals were then challenged with the inhalation of 1%w/v OVA solution in PBS for 30 min by a jet nebulizer on days 14, 15, and 16. Treatment started on day 12 for five consecutive days and lasted for 30 min (El-Kashef, 2018; van Wijck et al., 2018).

##### 2.2.12.3. Experimental design

**Figure 1.**
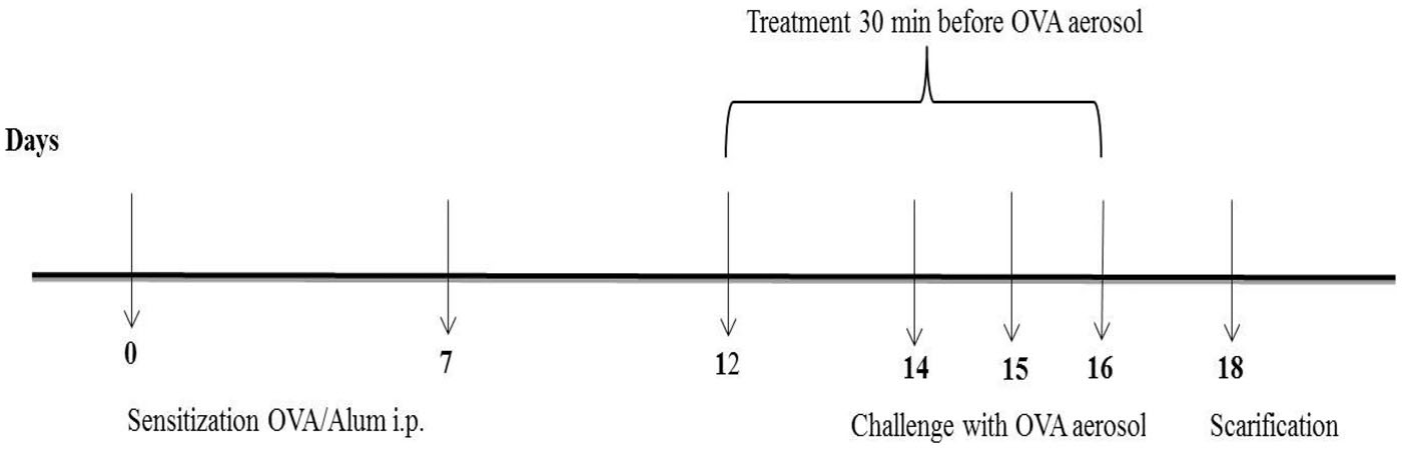
Experimental model for bronchial asthma induction and treatment scheme.

Mice were randomly divided into four experimental groups (*n* = 8); (A) control group: the mice were sensitized with i.p. Al(OH)_3_ on day 0 and 7 then challenged for 30 min with PBS aerosol. For treatment, the mice inhaled saline using nebulizer. (B) Sensitized group: this group underwent asthma induction and the treatment was the same as the control group. (C) CIC–NLP4 group: this group underwent asthma induction and the treatment was done using 40 µg CIC-NLPs. (D) Commercial CIC–MDI: this group underwent asthma induction and the treatment was with one puff of Alvesco^®^ (AstraZeneca, London, UK) 80 μg MDI for each mouse. Forty-eight hours after last challenge/treatment, the mice were anesthetized with i.p. ketamine (0.4 mg/g)/xylazine (0.2 mg/g) (Figure 1) and each group was divided into two subgroups, lungs from the first subgroups were used for collection of the bronchoalveolar lavage fluid (BALF), while lungs of the other subgroups were used for preparation of homogenates (left lungs) and histopathological examination (right lungs).

##### 2.2.12.4. Collection of bronchoalveolar lavage fluid

In the first subgroup, after anesthesia, the chest was opened, and trachea was cannulated. The lungs were then washed three times with 1.5 ml aliquots of ice-cold saline to collect the BALF. The fluid was centrifuged at 3000 rpm/4 °C for 5 min and the supernatant was rapidly stored at −80 °C for subsequent measurements of TNF-α, total and differential WBCs count (Yuting et al., 2019).

###### 2.2.12.4.1. Total and differential WBCs

Estimation of total and differential WBCs counts was done by resuspending BALF cell pellets using 5 ml cold PBS, then, total and differential cell counts as eosinophils, neutrophils, basophils, lymphocytes, and total leucocytes were analyzed by counting cells in at least five squares of a hemocytometer after excluding dead cells based on cellular morphology and staining characteristics (Da Cunha et al., 2016).

###### 2.2.12.4.2. Determination of TNF-α

The activity of TNF-α was determined in the BALF using a commercial enzyme-linked immunosorbent assay (ELISA) kit (MyBioSource, San Diego, CA) and its level was expressed as pg/g wet tissue.

##### 2.2.12.5. Preparation of lung homogenates

Left lungs of the second subgroup were harvested, weighed, and lung weight per body weight ratio was calculated. Then, lungs were homogenized in chilled 1.15% KCl (pH 7.45) using a tissue homogenizer (Biospec Products, Racine, WI) to yield 10% w/v tissue homogenates (Yuting et al., 2019). Homogenates were then used for the evaluation of interleukin (IL-4, IL-13), malondialdehyde (MDA), superoxide dismutase (SOD), and NO contents.

###### 2.2.12.5.1. Determination of IL-4 and IL-13

The activity of cytokines IL-4 and IL-13 in the lung homogenate were determined using commercial ELISA kits (CUSABIO Biotech Co., Ltd., Wuhan, China) according to the manufacturers' instructions after centrifugation at 3000 rpm for 20 min. Microtiter plates coated with anti-IL-4 and anti-IL-13 antibodies were incubated with lung homogenate supernatant. The plates were then washed several times as indicated and incubated for 10 min in the dark, then, the absorbance was measured at 450 nm and data were expressed as pg/g wet tissue.

###### 2.2.12.5.2. Determination of total lung nitrite/nitrate content

A concentration of NO in lung homogenate was measured using the Griess reagent. One hundred microliters lung homogenate supernatant was mixed with 100 μl of the Griess reagent and incubated at room temperature for 10 min. The absorbance was read at 540 nm according to a standard curve obtained from NaNO (Xu et al., 2018).

###### 2.2.12.5.3. Determination of MDA contents

Briefly, 100 µl aliquot of lung homogenate was added to reaction mixture containing 1.5 ml of 20%w/v trichloroacetic acid (pH 3.5) and 1.5 ml of 0.8%w/v thiobarbituric acid. Samples were heated at 95 °C for 30 min and cooled immediately then centrifuged at 5000 rpm for 10 min and the absorbance of supernatant was read at 650 nm (Impellizzeri et al., 2011).

###### 2.2.12.5.4. Determination of SOD

SOD activity was determined using the Marklund method (Marklund & Marklund, 1974). One hundred microliters of lung homogenate supernatant was added to 25 μl pyrogallol (24 mM pyrogallol in 10 mM HCl) and mixed with 2875 μl of 0.1 M Tris–HCl buffer (pH 7.8). Absorbance was measured at 420 nm and SOD activity represented as U/g tissue (El-Kashef & Serrya, 2019).

##### 2.2.12.6. Determination of IgE in serum

Serum IgE was determined using a specific mouse IgE ELISA assay kit (MyBioSource, Inc., San Diego, CA). Briefly, 96-well microtiter plates were coated overnight with anti-IgE antibodies and incubated with serum samples. The plates were then washed, o-phenylenediamine dihydrochloride was added to each well. After incubation for 10 min in the dark, absorbance was measured at 450 nm (Wijerathne et al., 2017).

##### 2.2.12.7. Western blot analysis

Total protein concentration in the homogenate was assayed using the Bradford reagent. Part of the homogenate was lysed using RIPA buffer with protease and phosphatase inhibitor mixture. Twenty-five micrograms of total protein were separated by 12% SDS-PAGE and transferred to polyvinylidene fluoride (PVDF) membranes. Membranes were incubated overnight at 4 °C with one of the following primary antibodies: total nuclear factor-κB (NF-κB)p65, phosphorylated NF-κBp65 (Cell Signaling Technology, Boston, MA) or β-actin (Thermo Fisher Scientific Inc., Waltham, MA). After washing, peroxidase-labeled secondary antibodies were added for 1 h and the band intensity was analyzed. The results are presented as arbitrary units after normalization to levels of the β-actin protein.

##### 2.2.12.8. Lung histopathology

Autopsy samples were taken from the right lung of mice in different groups for staining by hematoxylin and eosin stain for routine examination.

##### 2.2.12.9. Statistical analysis

The results are expressed as means ± SD. Statistical analysis was conducted using one-way ANOVA (Prism software, Version 6.05, GraphPad Inc., La Jolla, CA). The statistical significance was determined at *p*<.05.

## 3. Results and discussion

### 3.1. Lipids screening

**Figure 2.**
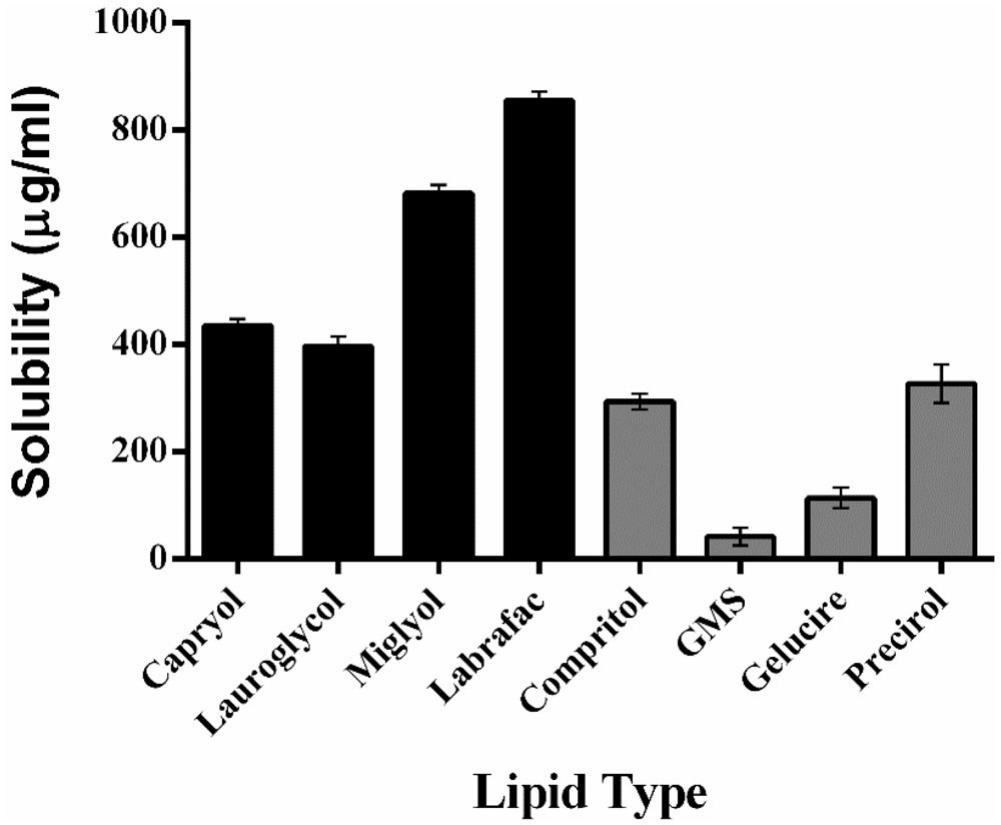
Solubility of CIC in various solid and liquid lipids (µg/ml).

The development of nebulized CIC-NLPs for the lung is a good alternative for improving the current ethanol based MDI treatment. The efficient CIC pulmonary delivery is considerably challenging by the rapid lung clearance and necessitates careful selection of lipids and surfactant to achieve safe suitable aerodynamically aerosolized nanoparticles for deep local alveoli deposition (Beloqui et al., 2016). All solid and liquid lipids used are biocompatible carriers of excellent safety profile and highly tolerable to the lung (Sanna et al., 2004; Pastor et al., 2014; Patil-Gadhe et al., 2014; Zhang et al., 2014; Chana et al., 2015; Moreno-Sastre et al., 2016; Huguet-Casquero et al., 2020). The solid lipids exhibited high melting point above 40 °C to prevent formation of super cooled melts so that the prepared NLPs remain in the solid state at room and body temperature (Mendes et al., 2013). Keeping in mind the lipophilic nature of CIC and its poor water solubility with log *p* value of 5.3, one can understand the highest CIC payload with maximum solubility in precirol (327.01 µg/ml) followed by compritol (293.96 µg/ml) as predicted from Figure 2. Although both of them are lipophilic triglycerides of long chain fatty acids (C18 and C22, respectively) with absence of PEG esters and low HLB value of ∼2 (Hamdani et al., 2003), the superior solubility of precirol stem from the difference in their crystalline properties where, the low tendency of precirol to crystallize into different polymorphic forms host more CIC (Shevalkar & Vavia, 2019) in contrast to highly crystalline compritol with perfect lattice (Huang et al., 2008).

One of the essential parameters to develop successful pulmonary NLPs of high drug payload is not only the selection of appropriate liquid lipid to form CIC enriched core and shell for prolonged pulmonary delivery but also the type of oil used can influence the pulmonary function. The addition of oil to the solid lipid, produces imperfections to the solid lattice structure thus, separating the chains of the fatty acids leaving more voids for CIC accommodation (Mubarak & Mohamed, 2018). Aside from long chain triglycerides (LCTs) oils that have been proven as crucial mediators of chronic airway inflammation with potential proinflammatory properties, that lead to impairment in pulmonary function (Sabater et al., 2011). Only medium chain triglycerides (MCTs) are used for CIC screening in this study. In particular, MCT of acrylic (C8:0)/caprice (C10:0) acids of better fluidity, higher solubilization capacity (Aburahma et al., 2010) and absence of double bonds that catalyze oxidation and elevate anti-inflammatory activation markers significantly (Yu et al., 2019). It was depicted from Figure 2 that labrafac and miglyol 812 provide greatest solubility for lipophilic CIC (855.09 ± 16.52 and 681.86 ± 15.69 µg/ml, respectively) than more hydrophilic propylene glycol ester based MCTs capryol and lauroglycol (435.48 ± 12.51 and 396.81 ± 18.11 µg/ml, respectively). Therefore, both (precirol and compritol) as well as (labrafac and miglyol 812) were selected and tested as solid–liquid lipid matrix for CIC-NLPs preparation.

### 3.2. Compatibility between binary lipid mixture

Among different tested solid–liquid lipid ratios (9:1, 8:2, 7:3, 6:4, and 5:5), only the ratios containing higher solid lipid content (9:1, 8:2, and 7:3) demonstrated excellent lipid miscibility with neither detected oil droplets on the filter paper nor drug recrystallization observed visually and under light microscope. Consequently, the effects of higher and lower oil concentrations (7:3 and 9:1) were only processed further for optimization of CIC-NLPs formation.

### 3.3. Preparation of CIC-NLPs

CIC-NLPs were prepared using inexpensive, easy scale-up, low-energy, hot microemulsion approach in which water-insoluble CIC was solubilized in the dispersed oil phase of the microemulsion, to obtain high CIC entrapment in the cured NLPs when the hot microemulsion cooled down to room temperature (Joshi & Patravale, 2006). Lecithin, a truncated cone of double-hydrophobic tails and zwitter ionic large head group phospholipid was used as a surfactant for CIC-NLPs preparation. In this regard, not only the safety, biocompatibility (Mendes et al., 2019), and potent beneficial lecithin function in attenuating leukocytic reactions and pro-inflammatory cytokines (Jung et al., 2013), but also, the hydrophobic nature of oil soluble lecithin (HLB∼8) is important for stabilizing O/W nanoemulsion droplets with low tendency to desorb into the aqueous continuous phase (Athas et al., 2014). Yet, lecithin alone is not adequate to stabilize the remarkable increase of the newly formed surface area during the transition of nanoemulsion droplets to form NLPs by cooling crystallization and solidification. The low mobility of the formed lecithin vesicles make it difficult to reach and cover the newly formed bare interfacial surface immediately and this necessitates the addition of extra hydrophilic co-surfactant of high water solubility (Han et al., 2008). Tween 80 was added to forms a reservoir of molecules that can be readily adsorbed to the interface to stabilize the naked newly formed interfacial region (Salminen et al., 2014). The three hydrophilic oxyethylene chains ‘hairs’ present on Tween 80 head group are extending to the aqueous phase, providing steric repulsive barrier between oil droplets leading to their stabilization and preventing their coalescence (Evans & Wennerstrom, 2001; Morrison, 2002). The subsidiary interactions that took place between hydrophobic tails of both Lecithin and Tween 80 through Van der Waals forces in addition to the hydrogen bonding that occurs between hydroxyls on Tween’s 80 head and phosphate groups on lecithin’s head. Due to the latter favorable interactions, lecithin and Tween 80 are closely backed to each other at the O/W interface thus, creating stable persisting interfacial film (Shchipunov & Shumilina, 1995). Moreover, lecithin and Tween 80 differ in their alkyl chain (tails) saturation degree. Tween 80 has one *cis* unsaturated oleic acid chain, whereas, lecithin possesses two C16–C18 tails, one of them has two *cis* unsaturations and the other tail is saturated (Shchipunov & Schmiedel, 1996). The existence of *cis* unsaturations assures that the tails remain liquid like at room temperature (Evans & Wennerstrom, 2001; Morrison, 2002). In contrast, the saturated tail tends to be rigid and solid at the same temperature which is vital for NLPs formation. We believe that all factors together demonstrate the synergy between lecithin and Tween 80 for the formation of stable CIC-NLP.

### 3.4. Statistical evaluation of the experimental factorial design for CIC-NLPs optimization

Table 1 presents 16 experimental runs for the prepared CIC-NLPs with their corresponding responses, while the statistical validations of the polynomial equations were established by ANOVA available in the software.

#### 3.4.1. Effect of formulation variables on PS and PDI

NLPs size is a key parameter that affects tissue penetration and cellular internalization. Particles <500 nm have efficient intracellular internalization and deep deposition while those larger than 500 nm are taken up by phagocytosis (Elena Fernández Fernández et al., 2018; Zhao & Stenzel, 2018). A wide PS distribution was obtained from 16 runs, ranging from 222.6 ± 4.52 nm (NLP1) to 1694 ± 13.07 nm (NLP9). The results revealed that all the investigated factors and their combined interactions have significantly contributed (*p*< .05) to PS in a direct proportional manner with high coefficient of determination value (*R*^2^=0.969) indicating a good model fit for the data except liquid lipid type (*p*> .05). The effect of independent variables on PS was illustrated in the following linear regression equation after eliminating the non-significant terms:

(4) Y1= +686.99+124.83A+232.3B+112.59C+101.34AB+134.82AC+57.32BC

where the positive and negative signs before a coefficient indicate a direct or inverse effect on the tested response, respectively (Elkady et al., 2020). It is evident that all formulae (NLP9–NLP16) prepared with high homogenization speed of 20,000 rpm have unfavorable large PS than those prepared using 10,000 rpm (NLP1–NLP8). This trend could be attributed to the system over processing where the additional energy input increases the knocking frequency between particles leading to coalescence and size enlargement (Anarjan et al., 2015). The 3D surface plot in Supplementary Figure S1(A–D) showed the prevalence of green area at higher speed indicating an increase in PS, while the grow of acceptable blue area with speed inclination to 10,000 rpm, indicates significant size reduction. Similarly, increasing liquid lipid concentration in (NLP 1, 3, 5, 7, 9, 11, 13, and 15) has significantly increased PS due to particles core swelling with liquid lipid (Granja et al., 2017). This is because at high temperature, there is a complete miscibility between melted solid and liquid lipids forming one phase. But during cooling process, and due to the miscibility gap between solid and liquid lipid, phase separation took place which is necessary for particles production and oil droplets precipitated inside the solid lipid matrix (Kovacevic et al., 2011). Furthermore, the glycerides alkyl chain length forming solid lipids can potentially affect PS. NLPs prepared using compritol are significantly (*p*= .021) of higher PS than those of precirol as shown in Supplementary Figure S1(A, B), where the prevalence of acceptable blue region was quite low when compritol was used indicating that the size was significantly increased. This is directly linked to the more hydrophobic compritol of longer chain length (C22) which hamper its dispersion in aqueous media and produce more viscous dispersion that reduce the homogenization efficiency in decreasing PS compared to short (C16–C18) more hydrophilic precirol (Durán-Lobato et al., 2016; Elmowafy et al., 2017). As a result, small PS below 500 nm was achieved in formulations NLPs 2, 3, 4, 6, and 8. Concurrently, PDI which measure the size distribution and the uniformity of the prepared NLPs is a fundamental attribute for their effective stability, efficacy, and high tissue accumulation (Danaei et al., 2018). Ideally, for a uniform monodisperse system, PDI value should be close to 0.0 whereas a value of less than 0.3 represents acceptable narrow size distribution (Ong et al., 2019). A wide PDI range was obtained for the prepared CIC-NLPs ranging from 0.053 ± 0.018 to 0.946 ± 0.033 (Table 1). ANOVA results revealed that, both homogenization speed (B) and solid lipid type (C) have significantly contributed (*p*= .0004 and *p*= .0161, respectively) to PDI in a direct proportional manner (Supplementary Figure S1(E–H)). This finding was illustrated in the following equation with good correlation of *R*^2^=0.946.

(5) Y2= +0.52+0.25B+0.11C

Similar to PS results, and in line with previous reports (El-Kamel et al., 2007; Anarjan et al., 2014), it is worth to note that, successful CIC formulations with smallest PDI could be achieved in NLP (3, 4, 7, and 8) prepared using precirol as solid lipid at low homogenization speed of 10,000 rpm only (Table 1).

#### 3.4.2. Effect of formulation variables on EE%

All the developed NLPs depicted successful CIC-EE% ranging from 73.968 ± 0.048% (NLP1) to 93.316 ± 0.24% (NLP4) as evident from Table 1. Both the lipophilic CIC nature associated with its high solubility in the selected lipids together with the voids created due to the imperfection caused by the combination of liquid and solid lipid were assigned to the improved EE% and accommodation of more CIC molecules (Mendes et al., 2019). ANOVA results revealed significant (*p*< .05) inverse relationship of solid: liquid lipid ratio (A) and solid lipid type (C) on EE% with no significant impact of their interaction was detected. This was illustrated in the following linear regression equation and graphically represented in Supplementary figure S1(I–L) with acceptable correlation of *R*^2^=0.914.

(6) Y3= +87.75−2.92A−2.11C 

Table 1 reveals that, the systems developed at higher oil ratio (7:3) showed lower EE% than those attained at lower level (9:1) (*p*= .0068) due to the expulsion of the excess oil that could not be accommodated by the solid lipid during crystallization process. When the liquid lipid concentration exceeds the holding capacity of the used solid lipid, the oil phase with the contained solubilized drug was ejected resulting in drug accumulation at the outer shell of the nanoparticles and thus reducing the EE% (Soleimanian et al., 2018). Moreover, the careful inspection of NLPs 1, 2, 5, 6, 9, 10, 13, and 14, endorsed that using compritol as solid lipid was concurrently decreasing EE% compared to precirol (*p*= .022). This result correlated well with the higher solubility of CIC in precirol and the presence of relative higher amount of mono-glycerides that possess surfactant properties (8–22%) (Abdel-Salam et al., 2017) than compritol (12–18%) (Kallakunta et al., 2018) which contribute in dissolving more drug and impart more looser and porous structure for CIC accommodation thereby explain its higher CIC entrapment and loading (Khames et al., 2019).

Therefore, in view of the aforementioned results, four NLPs 2, 4, 6, and 8 of PS <500 nm, PDI <0.3 and maximum EE% were processed for further *in vitro* release evaluation.

### 3.5. *In vitro* drug release

**Figure 3.**
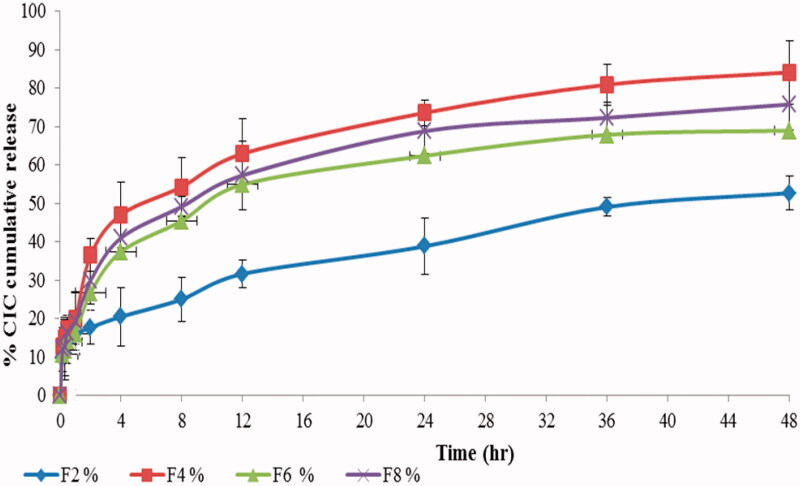
*In vitro* release profile of CIC loaded nanolipid particles (CIC-NLPs) in Gambleʼs solution of pH 7.4 containing 0.5%v/v Tween 80 at 37 °C (all points represent mean ± SD, *n* = 3).

The Franz diffusion cell apparatus provided a more discriminative profile in the current work than the conventional large volume dissolution apparatus that may lack a well *in vitro*–*in vivo* correlation (Elkady et al., 2020). CIC release profile followed a biphasic pattern characterized by an initial burst after 1 h (15.88–20.07%) followed by a controlled release for 48 h (Figure 3). The rapid cooling and disparity in melting behavior between solid and liquid lipid during NLPs preparation result in expulsion of oil and superficial DL in the outer layers of the NLPs (Lim et al., 2014). Therefore, increasing the amount of drug loaded on the outer shell as depicted in Table 1, would account for more drug released at the initial stage (NLP 4 > 8>2 > 6) (Huang et al., 2017). In the latter phase, the encapsulated CIC was released in a sustained manner over 48 h from deeper liquid and solid lipid cores by means of erosion and diffusion mechanisms (Almousallam et al., 2015). This combination of the burst and the sustained drug release is particularly beneficial for local treatment of bronchial asthma where, burst release will result in immediate pulmonary anti-inflammatory effect while, sustained release is expected to prolong CIC pulmonary residence time and increase its lung exposure thereby increasing the desired targeted pulmonary effects and limiting its penetration into systemic circulation and hence, diminish the drawbacks associated with CIC frequent dosing (Moreno-Sastre et al., 2016). On the other hand, the difference in cumulative drug release at the end of 48 h, could be arranged as follows: NLP4 > NLP8 > NLP6 > NLP2 with 84.07, 75.73, 68.90, and 52.67% CIC release, respectively. This behavior is related to the difference of entire composition of NLPs where the lower precirol melting point induces greater mobility (NLP 4, 8) than compritol (NLP 2, 6) during the experimental release temperature leading to drug diffusion from lipid core with significant increase in cumulative drug release (El-Housiny et al., 2018). Nevertheless, NLP4 release profile\was found to be significantly higher (*p*< .05) than NLP8 which mainly could be ascribed to NLP4 reduced PS that provides greater surface area.

In order to figure out the drug release mechanism, *in vitro* release profile for NLPs was fitted in different kinetic models (zero order, first order, Higuchi equations, Hixson–Crowell, and Korsmeyer–Peppas). A linear relationship between the amount of drug released and square root of time was obtained indicating Higuchi’s diffusion model (*R*^2^=0.934). Based on Korsmeyer–Peppas models, the magnitude of release exponent ‘n’ was found to be 0.618 within the range of 0.85>*n* > 0.43, which implies that, CIC release obeys non-Fickian diffusion mechanism (*R*^2^=0.973), depending on the degradation of the lipid chain rather than water penetration into the matrix (Gonzalez-Mira et al., 2011).

Consequently, based on the optimization of the factorial design and the desirability function evaluated using Design-Expert software, NLP4 showed the highest desirability index (*D* = 0.964) with the best attributes of the lowest PS (222.6 nm) and PDI (0.23), maximum EE (93.3%), and highest CIC release of 84% after 48 h.

### 3.6. TEM and zeta potential analysis

The morphology of NLP4 analyzed using TEM revealed spherical, well-identified discrete nanoparticles with PS correlates well with the size obtained from PCS (Supplementary Figure S2), with a desirable negative high ZPs of −40.0 mV necessary for adequate repulsion between the nanoparticles, thus preventing their aggregation and ensures a long-term physically stable system (Yousry et al., 2017). The acquired –ve charge is very advantageous for the safe internalization of NLPs into the cells by endocytosis, avoiding cytotoxicity and detrimental impact commonly induced by positively charged particles on the cell membranes (Zhao & Stenzel, 2018). The lecithin anionic phospholipids such as phosphatidyl serine and phosphatidyl glycerol (Luo et al., 2017) as well as the slightly ionized Miglyolʼs fatty acids (Sanad et al., 2010) were adsorbed and accumulated at the oil droplet surface and were responsible for imparting NLPs –ve charges.

### 3.7. *In vitro* lung deposition study

Depending on PS, the deposition of aerosol particles at each stage of ACI is expected to mimic the deposition patterns at various regions of the human lung from upper airways to alveolus (Ali, 2010). Alveolar deposition is optimal with aerodynamic PS of 2–3 μm (Said-Elbahr et al., 2016), while particles >5 µm are trapped in the upper airways and those less than 0.5 µm are exhaled and fail to deposit (Silva et al., 2017).

The *in vitro* aerosolization pattern of CIC–NLP4 following nebulization via ACI operating at flow rate of 28.3 l/min was assessed. MMAD and GSD that reflects the spread of the aerodynamic particle distribution (Arbain et al., 2018) as well as FPF% (the respirable fraction that is most likely to deposit in the deep lung) (Silva et al., 2017) were all determined and used as the key aspects to characterize the respirability of the developed CIC-NLPs dispersion.

**Figure 4.**
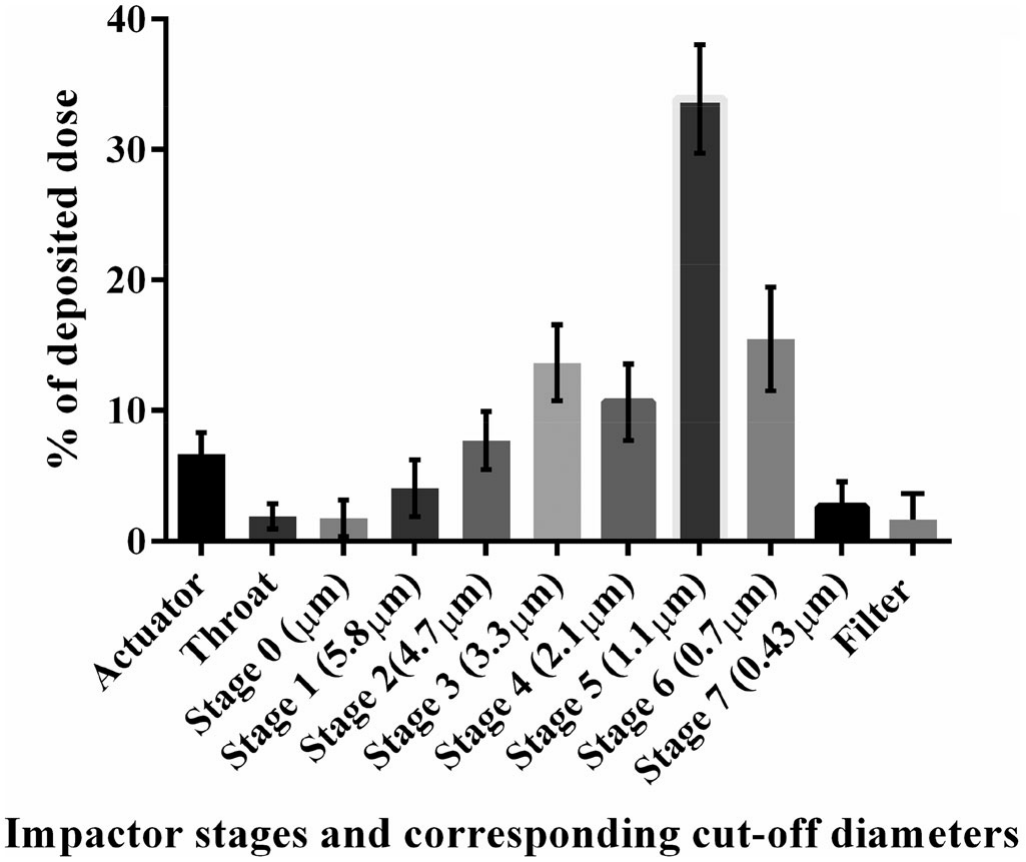
*In vitro* aerosol performance of nebulized CIC–NLP4 at 28.3 l/min using Anderson 8-stage non-viable cascade impactor.

It was perceived from Figure 4 that, the total amount of CIC deposited in all ACI stages from stage 2 to final filter was ∼87% (compared to ∼61% of commercial preparation) (Newman et al., 2006). Herein, the highest proportion and the majority (∼50%) of CIC–NLP4 emitted dose were successfully deposited in the lower stages, stage 5 with 33.88 ± 4.17% deposition and stage 6 with 15.48 ± 3.98% representing the peripheral region including small airways and alveoli (Arbain et al., 2018). On the contrary, despite the small aerodynamic PS of the commercial CIC–MDI and the use of aero-spacer to minimize the oropharynx deposition, all previous HFA CIC–MDI reports revealed consistent drug loss of ∼35–40% due to the induction port-mouth piece deposition (Boulet et al., 2006; Newman et al., 2006; Nave & Mueller, 2013) compared with negligible deposition of only 8% of the nebulized CIC–NLP4. The impaction results revealed an ideal measured MMAD of 2.03 ± 0.19 µm which was well correlated with the standard recommendations and optimal properties for small airways aerosolization (Corren & Tashkin, 2003; Douafer et al., 2020). Not only MMAD is important, but also GSD (which is one standard deviation above and below the MMAD) could greatly affect the therapeutic effectiveness of the developed inhaled CIC dispersion. The determined GSD was 2.3 ± 0.24 indicating broad aerodynamic diameter of CIC-NLPs around the mean MMAD, thus allowing them to be dispersed at different impactor stages. This profile could be of considerable therapeutic advantage for asthma treatment where, asthma patients could benefit from the possible targeting of the entire airway tree where the treating of the small airway region by poly disperse CIC-NLPs was achieved (Lavorini et al., 2017). In addition to MMAD and GSD, FPF% was used to gauge the efficiency of lung deposition (Bharatwaj et al., 2010). The developed CIC–NLP4 revealed higher FPF% of 84.51 ± 5.1% exceeding the commercial CIC–MDI (FPF of 54%) (Drollmann et al., 2006) and the previous reported exclusive nebulized CIC study (FPF of 43.3%) (Fu et al., 2018). Therefore, the aerosolization ACI results evidenced higher FPF% accompanied by lower MMAD and GSD, minimal oropharyngeal deposition and greater dose delivery to the lung than MDI which supports the developed CIC–NLP4 as efficient treatment of bronchial asthma since it possesses all the features to render it inhalable with deep lung deposition.

### 3.8. DSC analysis

Supplementary Figure S3 displays DSC thermograms obtained for pure CIC, NLP4 physical mixture, and CIC–NLP4. Pure CIC showed sharp melting endothermic peak at 210 °C which was retained in its physical mixture indicating the persistence of drug crystalline nature and the lack of any interactions. When the components were processed as NLPs, the thermogram of CIC–NLP4 revealed the disappearance of CIC characteristic peak with shifting of lipid phase peak from 62 °C to 124 °C when compared to the physical mixture. This would confirm the presence of the drug in its amorphous state and the distortion of the lipid matrix crystalline nature due to the presence of liquid lipid and surfactants inside the lipid matrix leading to crystal lattice defects during phase transition to support drug accommodation (Salem et al., 2020).

### 3.9. *In vitro* cytotoxicity assay

**Figure 5.**
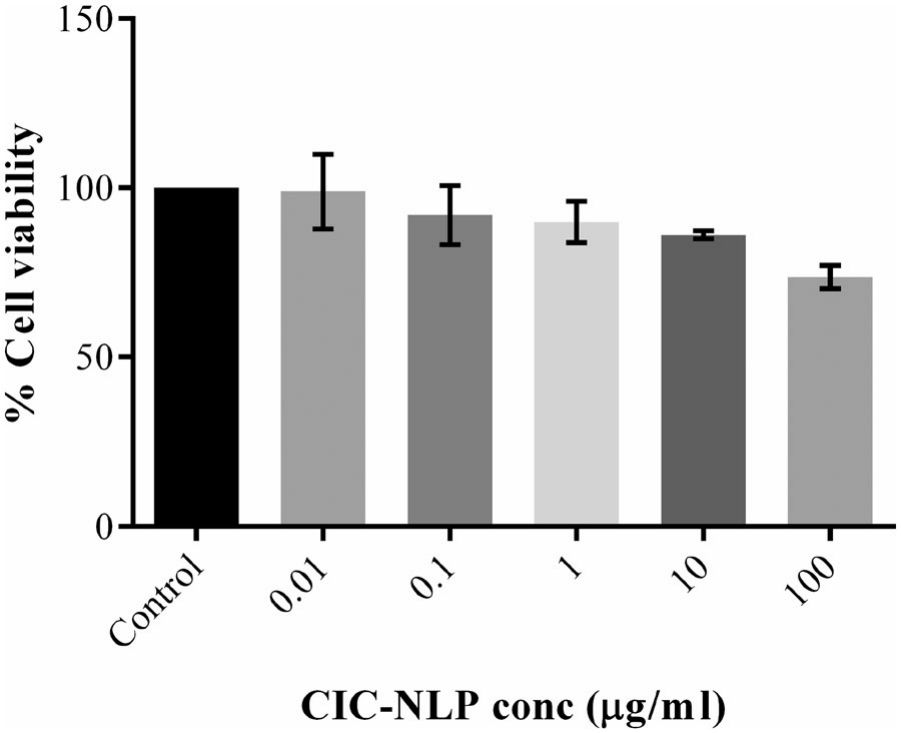
Effect of exposure of CIC–NLP4 with different concentrations of CIC on the viability of A459 cells determined by SRB assay. Each value represents the mean ± SD (*n* = 3).

The toxicity profile of the optimized CIC–NLP4 is influenced by the nature and the type of lipids, surfactants, drug used as well as their proportion and concentration in the formulation (Guilherme et al., 2019). The safety and efficacy results performed on human epithelial cell line A549 after incubation for 72 h are illustrated in Figure 5. No evidence of cell viability reduction by more than 30% was noticed and all cell survival values exceed 70% at all tested concentrations (∼74%±1.18% for the highest concentration of 100 μg/ml) denoting well tolerated formulation (Gartziandia et al., 2015; Levy et al., 2019) and considering CIC–NLP4 as cytocompatible without imparting cytotoxic effect up to a concentration of 100 μg/ml. Therefore, the lipids used with the optimum Tween 80 concentration are safe to achieve small size, biocompatible, and nontoxic CIC-NLPs without cell damaging effect of the excess unbound surfactant (Jana et al., 2016). Hence, CIC–NLP4 was progressed to further *in vivo* study to investigate its efficacy against allergic airway inflammation.

### 3.10. *In vivo* study

#### 3.10.1. Effect of CIC–NLP4 on asthma markers in OVA-challenged mice

Induction of asthma resulted in higher counts of these inflammatory cells in BALF, associated with a significant increase in the lung weight index by about 82.22% compared to normal control group (*p*< .05) indicating pulmonary edema with accumulation of serous fluids within the lungs due to the inflammatory action. Treatment with nebulized CIC (NLP4 and commercial CIC–MDI led to fewer lung lavage inflammatory cells than in positive control mice (Table 2).

**Table 2.** Effect of CIC on total and differential cell counts in BALF of mice, expressed as mean values ± SD.

Group	Total leukocytes	Lymphocytes	Eosinophils	Basophils	Neutrophils
Control	8175 ± 639.6	21.25 ± 1.71	1.75 ± 0.96	0	40.75 ± 2.22
OVA	16,950 ± 341.6	35.25 ± 2.22	6.5 ± 1.29	1.5 ± 0.58	64 ± 4.55
CIC–NLP4	10,075 ± 221.7*	26.0 ± 2.16*	2.25 ± 0.50	0.25 ± 0.5	47.75 ± 2.22*
CIC–MDI	10,950 ± 264.58	29.75 ± 0.96	3.0 ± 0.82	0.25 ± 0.5	52.5 ± 1.29

* Significant difference between CIC–NLP4 and CIC–MDI, *p*<.05.

CIC–NLP4 produced an anti-inflammatory activity that lasted for 48 h compared to CIC–MDI (starting from the last day of treatment), as the decline in cellular count was more significant in this group. Furthermore, CIC–NLP4 revealed a greater decline in the number of neutrophils, an aspect common in severe steroid-resistant asthma. This enhanced effect of CIC–NLP4 may be due to the ability of NLPs to enter and accumulate within the inflamed tissue thus, acting more efficiently (Matsuo et al., 2009).

Both treatment groups decreased lung weight index when compared to the OVA-challenged group by about 20% and 13%, respectively. Although the decrease was not significant, it can be attributed to the decreased inflammation-associated edema (Supplementary Figure S4).

The Th2 cytokines IL-4 and IL-13 are critical to the initiation and potentiation of the airway inflammation and drivers of the remodeling response including mucous production, smooth muscle cell hypertrophy, and epithelial cell proliferation (Kenyon et al., 2013). IL-4 enhanced the recruitment of eosinophils and basophils and switches B lymphocyte cells to the production of IgE which in turn, activates mast cells and basophils to release histamine and different cytokines in the bronchi. This explains the rise in the level of IgE in the serum of the OVA-group (71.3 ± 6.59 ng/ml).

IL-13 in turn, initiates changes in airway epithelial, goblet, and smooth muscle cells that define the chronic remodeling response through activation of the JNK and STAT6 (signal transducer and activator of transcription 6) pathways. CIC inhibits these pathways by inhibiting the translation and surface expression of IL-4Rs and preventing the action of JNK on STAT6 along with inhibiting the production of IL-4 and 13 (Yoshihiko et al., 2012). OVA induced a significant elevation of IL-4 and IL-13 concentration in the lung homogenate. Our BALF showed that IL-4 and IL-13 were significantly lower in the CIC treated groups compared to the OVA group (33.52% and 27.47% for CIC–NLP4 and 49.1% and 48.56% for CIC–MDI, respectively).

**Figure 6.**
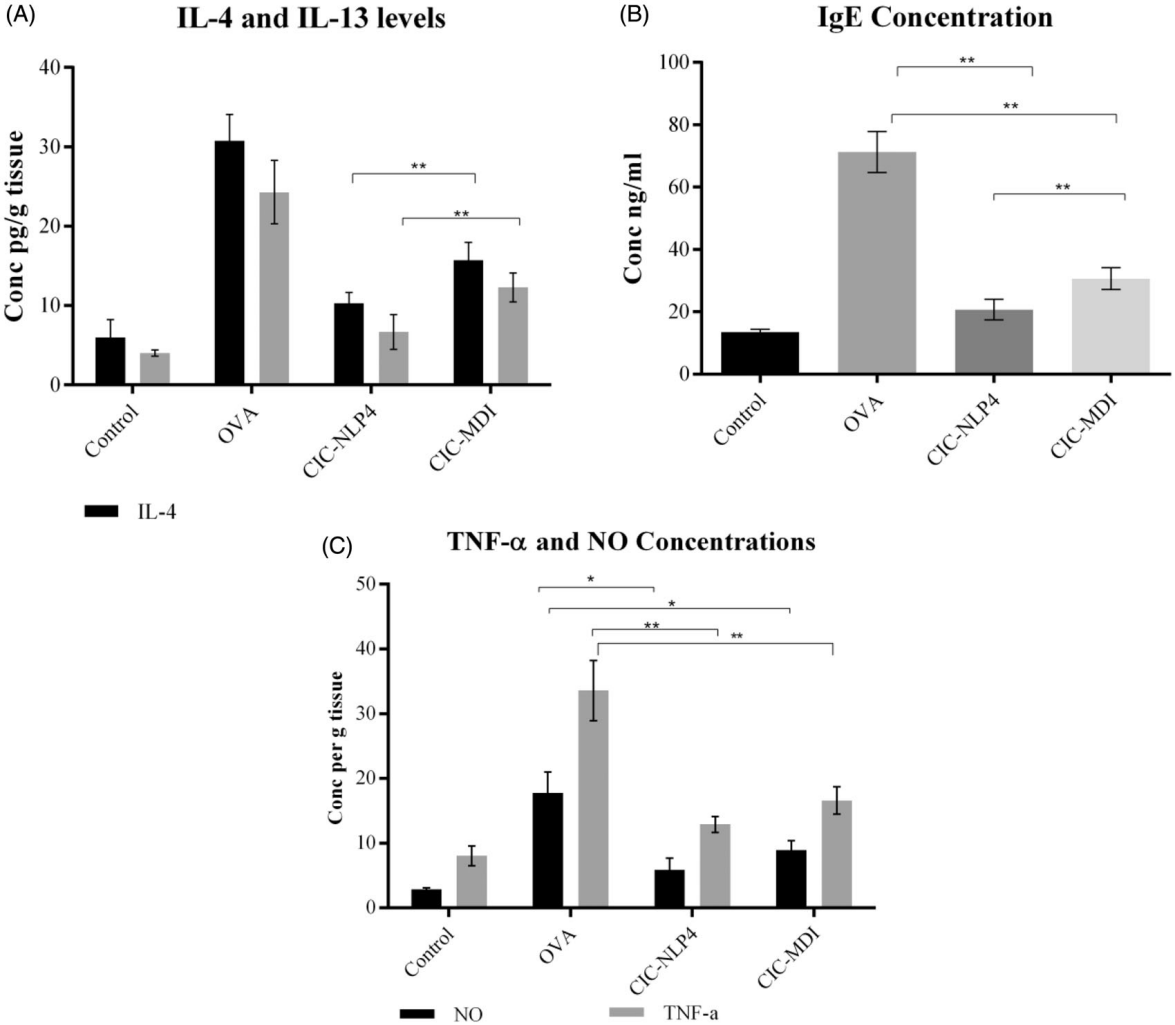
Effect of inflammatory markers in OVA-challenged mice. (A) Cytokines (IL4 and 13), (B) effect of CIC on serum IgE, and (C) effect of CIC on BALF TNFα and lung homogenate. *Significantly different (*p*<.05), **significantly different (*p*<.001).

CIC–NLP4 decreased the levels of IL-4 and IL-13 by 15.6% and 21.1% (Figure 6(A)) and IgE by 14% more compared to CIC–MDI (Figure 6(B)). Despite the lower dose, CIC–NLP4 was able to produce better anti-inflammatory effects than CIC–MDI. This can be attributed to the nanoparticle protection of CIC from the enzymatic degradation in the airways enabling a more potent Th2 lymphocyte inhibitory effect, better lung deposition, and the prolonged release of the drug (Kenyon et al., 2013).

#### 3.10.2. Effect of CIC–NLP4 on TNF-α and NO

Nitric oxide (NO) is produced by several cell types in the respiratory tract including epithelial cells, airway nerves, inflammatory cells, and vascular endothelial cells. It is an important mediator implicated in the progression of asthma where, extensive production of NO is cytotoxic and produces marked pathological alterations including inflammatory cellular infiltration, mucus production, and airway edema during asthma exacerbations (El-Kashef, 2018). Inducible nitric oxide synthase (iNOS) enzyme is responsible for the synthesis of NO and is regulated at a pre-translational level where it can be induced by pro-inflammatory cytokines such as TNF-α (Ricciardolo, 2003). In the current study, NO was increased fivefold and TNF-α by threefold in the diseased group compared to the control group (Figure 6(C)). CIC represses TNF-α signaling (Sasse et al., 2016) and decreases NO through the binding to glucocorticoid receptors and inhibiting its iNOS transcription (Griffiths et al., 2017).

CIC–NLP4 significantly decreased the levels of BALF TNF-α and NO compared to CIC–MDI. CIC–NLP4 showed 22% lower levels of TNF-α and 34% lower levels of NO than the CIC–MDI. We hypothesize that this improvement is due to lecithin used in the CIC–NLP4 formula. It has been reported that lecithin possesses an anti-inflammatory effect and can lower the levels of TNF-α (Jung et al., 2013) either by inhibiting the neutrophil leukocyte-mediated microcirculatory inflammatory reactions (Hartmann et al., 2009) and by inhibiting the transcription of iNOS and reducing the NO concentration (Erõs et al., 2009).

#### 3.10.3. Effect on oxidative stress biomarkers

**Figure 7.**
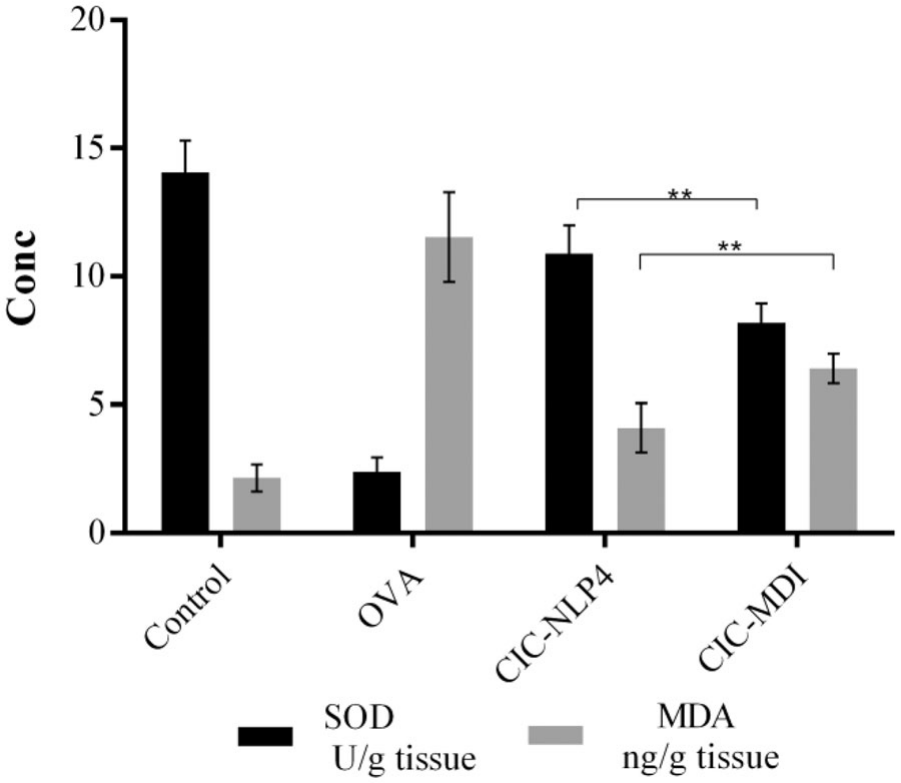
Effect of CIC on the oxidative stress biomarkers, MDA, and SOD in lung homogenate of OVA-challenged mice ± SD. **Significantly different (*p*<.001).

OVA significantly increased lung MDA content, an index of lipid peroxidation, by about fivefold in comparison with control group (11.5 and 2.25 ng/g tissue, respectively) with significant decrease in SOD activity by about sixfold (*p*<.05) (Figure 7). Inhalation of CIC in both treatment groups significantly decreased OVA-induced increase in MDA content to 35.6% and 55.5%, respectively. Moreover, both groups significantly increased SOD activity when compared with OVA-challenged group by about 4.5- and 3.4-fold, respectively (*p*<.05) with improvement of CIC–NLP4 group over CIC–MDI.

Oxidative stress contributes in asthma via activation of the NFκB pathway and enhancing the activation of eosinophilic extracellular traps (Silveira et al., 2019). Besides protein damage to several enzymes, including SOD (Comhair et al., 2005), oxidative stress results in the hyper phosphorylation and inhibition of HDAC2 enzyme resulting in the development of corticosteroid-resistant asthma (Chung & Marwick, 2010). Our study corroborates with other studies reporting an increase in oxidative stress markers in OVA-induced asthma (Nadeem et al., 2005; Zeng et al., 2013).

In the present study, we hypothesize that rapid onset of action in both formulations decreased cellular inflammation and thus, decreased the oxidative stress and MDA. In CIC–NLP4 group, there was a significant decrease in lung MDA content when compared with MDI. We attribute this to the use of Tween 80 and lecithin in our formula. Tween 80 (Pérez-Rosés et al., 2015) and lecithin (Pan et al., 2013) were reported to have antioxidative properties that can be useful in aiding healing injured tissue (Nasab et al., 2019). Bao et al. went for the potential use of lecithin for the treatment of airway inflammatory diseases due to its potent antioxidant effect (Bao et al., 2011).

Tween 80 in CIC–NLP4 was also reported to inhibit P-glycoprotein (Li-Blatter et al., 2009), a drug efflux pump encoded by the multidrug-resistance gene 1 that transports glucocorticoids out of the cells thereby, reducing the intracellular glucocorticoid concentration and decreasing the efficiency of treatment despite the high concentration of glucocorticoids (Xu et al., 2017). This improved effect of Tween 80 may account for the prolonged activity of our formula explaining greater efficiency of CIC–NLP4 over CIC–MDI despite the smaller dose of our formula.

#### 3.10.4. Western blot analysis

**Figure 8.**
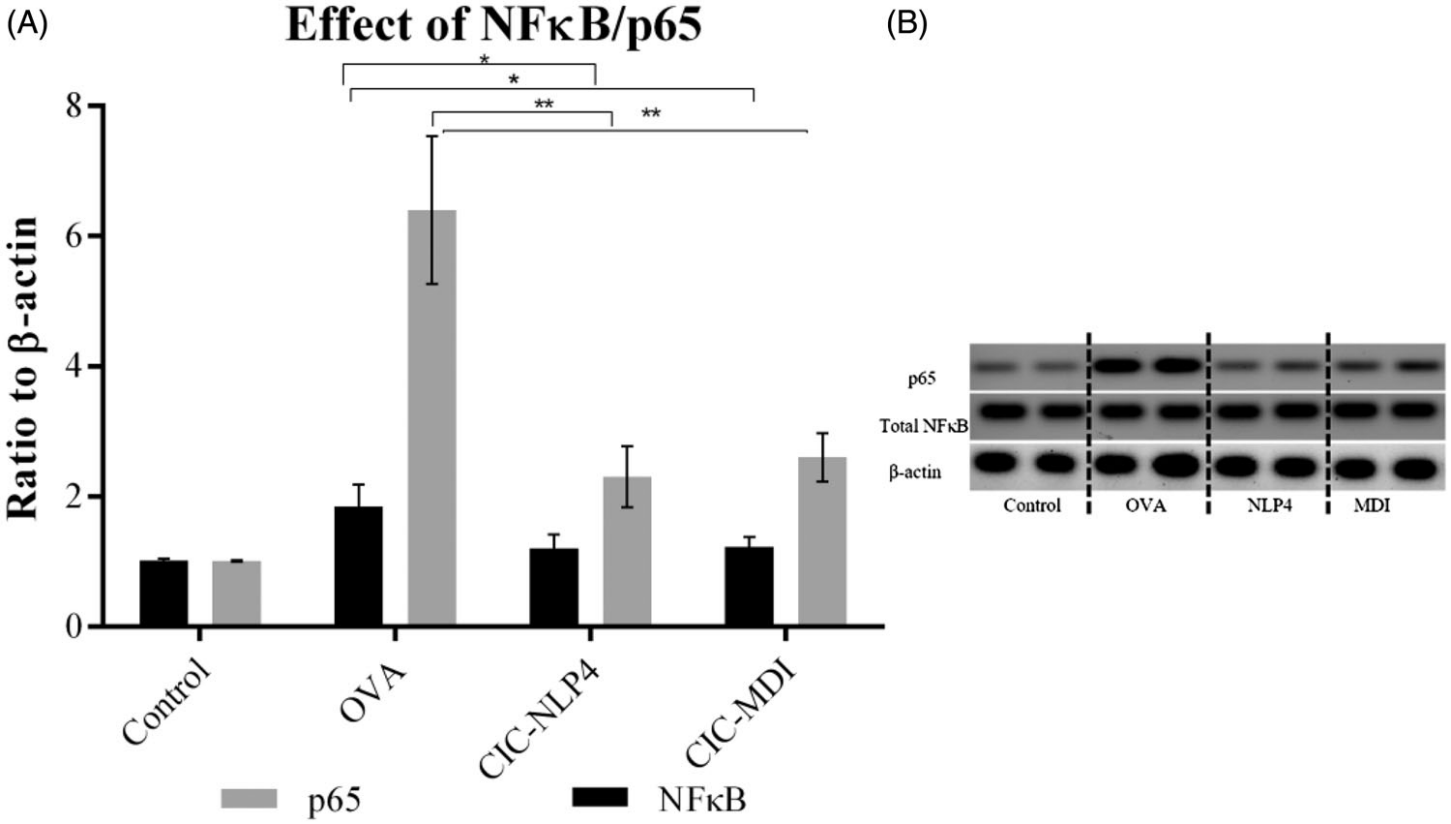
Effects of CIC on total NF-κB/p65. (A) Western blotting and (B) ratio to β-actin ± SD. *Significantly different (*p*<.05), **significantly different (*p*<.001).

Phosphorylated NF-Κb65 is a transcription factor responsible for upregulating pro-inflammatory genes including TNF-α (Barnes, 2016). The OVA-induced asthma resulted in the elevation of phosphorylated p65 by more than sixfold compared to the control group. This increase was diminished by CIC where, CIC–NLP4 was able to reduce the phosphorylated p65 by 64% which was significantly lower than CIC–MDI (Figure 8). We believe that lecithin played a role in inhibiting the activation of NFκB as was reported by others (Cheng et al., 2018; Choudhary et al., 2019). Treede et al. proposed a mechanism through which the effect on NFκB is through preventing its activation via TNF-α by causing a shift of the TNF-α receptors at the membrane surface to lipid rafts (Treede et al., 2009).

#### 3.10.5. Effect of CIC–NLP4 on lung histopathology

**Figure 9.**
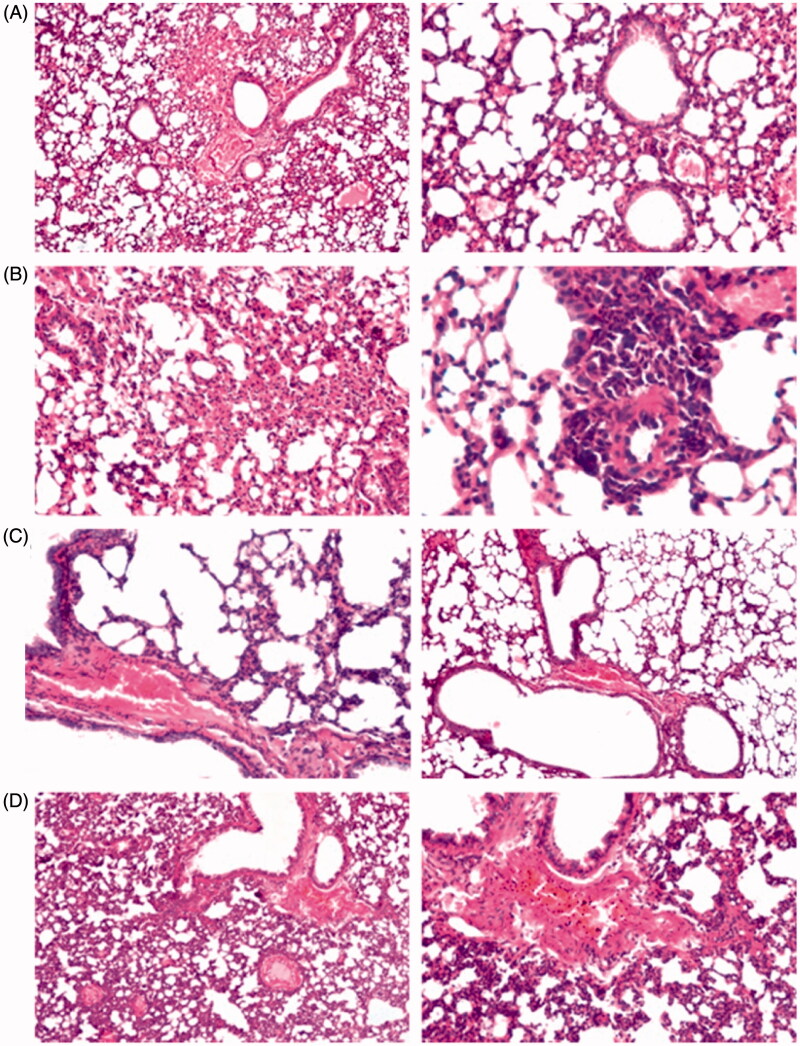
Representative histopathological changes in lungs obtained from mice of different groups showing the effect of CIC on: (A) control, (B) OVA-group, (C) NLP4, and (D) MDI.

To evaluate the effects of CIC on the histopathology of OVA-induced allergic asthma, histological changes of lung tissues with and without CIC treatment were detected by H&E staining (Figure 9). In the control group, there was no histopathological alteration and normal histological structure of the bronchiole as well as the peribronchiolar blood vessels and air alveoli were observed. In contrast, all OVA-sensitized and challenged mice showed peribronchiolar tissue with focal inflammatory cells infiltration as well as congestion in the blood vessels. Focal collapse was observed in the air alveoli associated with perivascular inflammatory cells aggregation indicating that our asthma model was well established. However, treatments with CIC remarkably ameliorated OVA-induced lung injury and inflammatory cells infiltration despite the presence of congestion in the peribronchiolar blood vessels and diffuse emphysema in the air alveoli. This improvement confirms the deep deposition of the drug as was previously proven by the *in vitro* lung deposition experiment. The insignificance of improvement between the two formulations is most probably due to the short treatment period although it was considered a great advantage to CIC-NLPs as the same effect was obtained at a lower dose. The relatively higher efficiency of CIC–NLP4 in preventing asthma inflammation over CIC–MDI may be attributed to the encapsulation of CIC in NLPs forming spherical shape and small nanoparticle size below 260 nm that can escape macrophage lung clearance (Patlolla et al., 2010) inducing deep drug deposition in a controlled release manner.

## 4. Conclusions

Owing to the prevalence of asthma, the present study explored safe, convenient nebulization mode for lung delivery of CIC. Lipid nanoformulation with favorable specific inhaled characteristics was developed to deliver CIC deep to the small airways, the inflammation target at lower dose, reduced oropharyngeal deposition, and prolonged release manner to avoid the incidence of side effects associated with ICSs. Most importantly, the merits of the developed nebulizable CIC–NLP4 display superior *in vitro* and *in vivo* properties when compared with the commercial CIC–MDI therapy. The successful high CIC payload together with the potential anti-inflammatory and antioxidative effects of the combined surfactant use of Tween 80–lecithin were behind the proved significant efficacy in the mitigation of the allergic airways inflammations and the magnitude of therapeutic cure using only 40 µg, half CIC commercial MDI dose. Overall, the aerosol properties obtained in this work (high FPF% and deep lung deposition) together with the sustained release profile encourage the once daily therapeutic use of CIC at half its commercial dose and hold great promise in the therapeutic modality of bronchial asthma. Consequently, future clinical studies on asthmatic patients are recommended.

## Supplementary Material

Supplemental Material
