# Supplementary material for: Enhanced alveo pulmonary deposition of nebulized ciclesonide for attenuating airways inflammations: a strategy to overcome metered dose inhaler drawbacks
Source: Drug Deliv. 2021 Apr 30;28(1):826–43. doi: 10.1080/10717544.2021.1905747 (PMC8812587; doi:10.1080/10717544.2021.1905747)
Supplement: Supplemental Material [file IDRD_A_1905747_SM0229.docx]

***Supplementary Data to accompany the manuscript:***

**Enhanced Alveo Pulmonary Deposition of Nebulized Ciclesonide for Attenuating Airways Inflammations: A strategy to Overcome Metered Dose Inhaler Drawbacks**

**Hanan M El-Laithy^1,2^ , Amal Youssef ^3^, Shereen S. El-Husseney^3^, NesreenSalah^1^, Ahmed Maher^2^**

^1^Department of Pharmaceutics and Industrial Pharmacy, Faculty of Pharmacy, Cairo University, Cairo, Egypt

^2^Department of Pharmaceutics and Industrial Pharmacy, Faculty of Pharmacy, October University for Modern Sciences and Arts (MSA), Cairo, Egypt

^3^Department of Pharmaceutics, Egyptian Drug Authority, Cairo, Cairo, Egypt

**Table S1: Gamble´s solution composition**

| Ingredients | Concentration  (g/l) |
| --- | --- |
| Magnesium chloride | 0.095 |
| Sodium chloride | 6.019 |
| Potassium chloride | 0.298 |
| Disodium hydrogen phosphate (Na2HPO4) | 0.126 |
| Sodium sulfate | 0.063 |
| Calcium chloride dihydrate | 0.368 |
| Sodium acetate | 0.574 |
| Sodium hydrogen carbonate (NaHCO3) | 2.604 |
| Sodium citrate dihydrate | 0.097 |


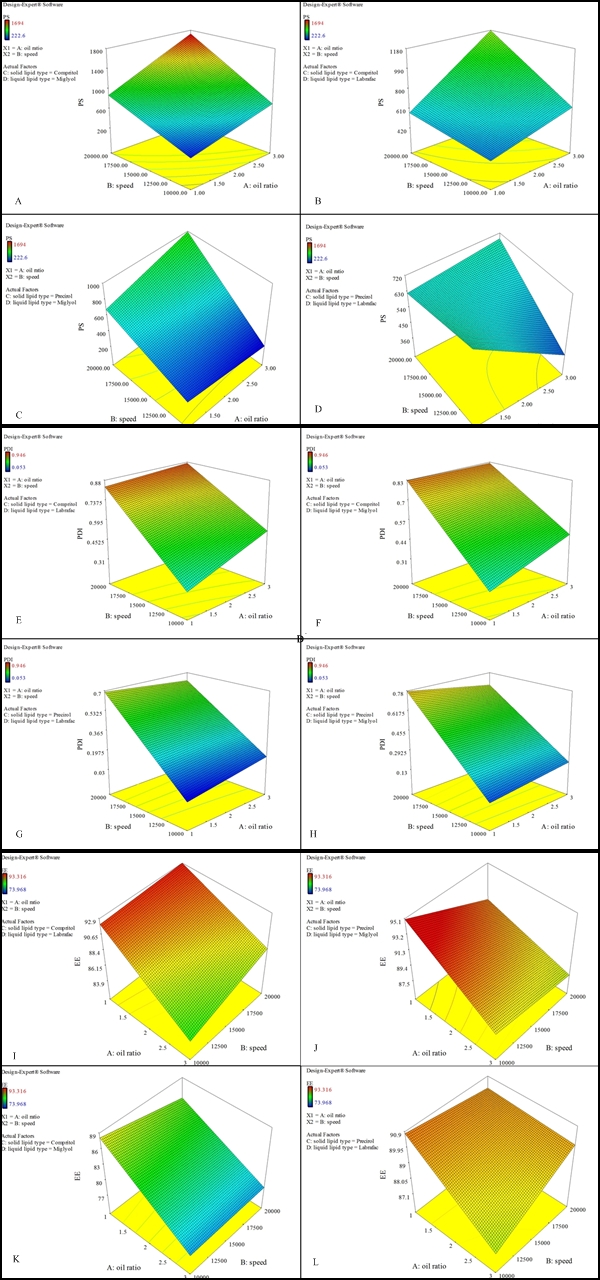


Figure S1: 3D response surface plot for the effect of the independent variables on particle size, PDI and entrapment efficiency


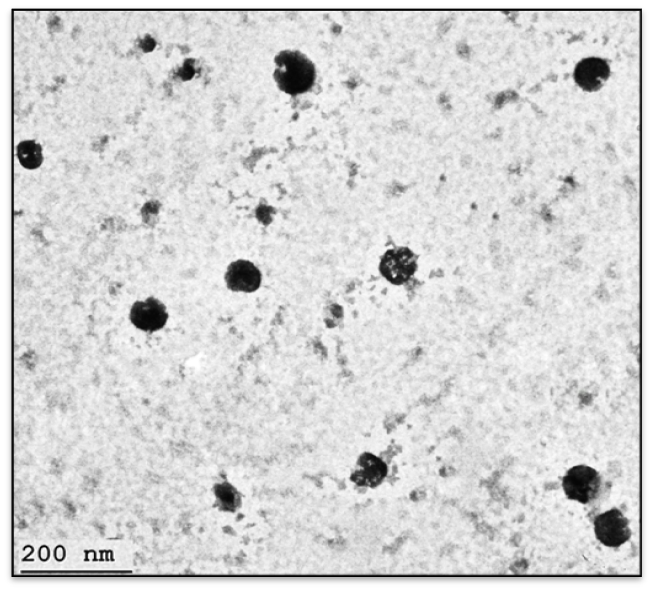


Figure S2: TEM micrograph of CIC loaded nanolipid particles (CIC-NLP4)


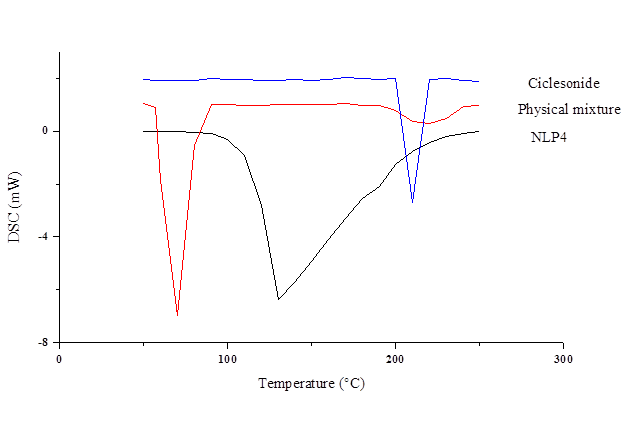


Figure S3: DSC thermograms of pure CIC, physical mixture and CIC-NLP4

**
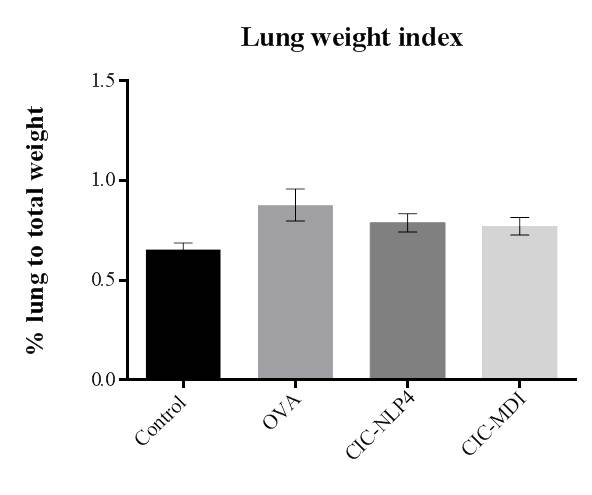
**

**Figure S4: Effect of CIC on the lung weight index of OVA-challenged asthma mice**
